# Multisystem Inflammatory Syndrome in Adults after Mild SARS-CoV-2 Infection, Japan

**DOI:** 10.3201/eid2706.210728

**Published:** 2021-06

**Authors:** Yasuhiro Yamada, Kaoru Fujinami, Tadashi Eguchi, Hiroshi Takefuji, Nobuaki Mori

**Affiliations:** National Hospital Organization Tokyo Medical Center, Tokyo, Japan (Y. Yamada, K. Fujinami, T. Eguchi, H. Takefuji, N. Mori);; University College London, London, UK (K. Fujinami)

**Keywords:** COVID-19, coronavirus disease, SARS-CoV-2, severe acute respiratory syndrome coronavirus 2, viruses, respiratory infections, zoonoses, multisystem inflammatory syndrome, Japan

## Abstract

In Japan, a 51-year-old man had minimally symptomatic severe acute respiratory syndrome coronavirus 2 infection. Multisystem inflammatory syndrome was diagnosed ≈5 weeks later; characteristics included severe inflammation, cardiac dysfunction, and IgG positivity. Clinicians should obtain detailed history and examine IgG levels for cases of inflammatory disease with unexplained cardiac decompensation.

Over the course of the coronavirus disease pandemic, severe inflammatory syndromes have been reported in children (*1**–**3*). Since June 2020, the same syndrome has also been reported in adults. The Centers for Disease Control and Prevention has been collecting case reports of multisystem inflammatory syndrome in adults (MIS-A) and published a case series of MIS-A reported from the United Kingdom and United States in November 2020 (*4*).

A healthy 51-year-old man in Japan tested positive for severe acute respiratory syndrome coronavirus 2 (SARS-CoV-2) by PCR on a saliva sample after his wife was infected with SARS-CoV-2. The positive result was obtained 37 days before hospital admission. During the course of his SARS-CoV-2 infection, his only symptom was olfactory disturbance; he had no respiratory symptoms or fever. He became aware of swelling in the right side of his neck and fatigue 3 days before admission. He visited an internal medicine clinic 2 days before admission for sore throat and fever in the range of 38°C and was prescribed levofloxacin for pharyngitis.

He initially came to the emergency department of National Hospital Organization Tokyo Medical Center because of fever and sore throat, which did not improve. On examination, we noted enlargement of the right cervical lymph nodes, and cervical contrast-enhanced computed tomography revealed lymph nodes swollen to 20 mm localized in the right side of the neck and swelling of the posterior wall of the middle pharynx. The patient was admitted with a diagnosis of lymphadenitis, and we initiated ampicillin/sulbactam.

The patient became acutely hypotensive with blood pressure of 73/45 mm Hg 2 days after admission. He was treated with noradrenaline and dobutamine, but blood pressure did not increase despite crystalloid fluid infusion. We changed antibiotics to meropenem and vancomycin, and 100 mg hydrocortisone was administered empirically to treat septic shock. An electrocardiogram showed a negative T wave and sinus tachycardia. Echocardiography showed ejection fraction of 42% and overall decreased left ventricular contraction. No pericardial effusion was observed. Systemic computed tomography showed enlarged lymph nodes only in the right side of the neck and no pneumonia in the lung fields. The patient was admitted to the intensive care unit (ICU) (Table).

**Table:** Laboratory studies performed at intensive care unit admission of patient with multisystem inflammatory syndrome after mild severe acute respiratory syndrome coronavirus 2 infection, Japan

Laboratory test	Result	Reference range
C-reactive protein, mg/dL	36.77	<0.14
Procalcitonin, ng/mL	3.67	<0.05
Interleukin 6, pg/dL	565	<4
Leukocyte count, × 10^9^ cells/L	22.4	3.0–8.6
Neutrophil count, × 10^9^ cells/L	21.0	1.5–5.8
Lymphocyte count, × 10^9^ cells/L	1.0	1.0–3.0
Hemoglobin, g/dL	13.2	13.7–16.8
Platelets, × 10^9^/L	180	158–348
Serum creatinine, mg/dL	2.54	0.65–1.07
Albumin, g/dL	2.5	4.1–5.1
Aspartate aminotransferase, U/L	19	13–30
Alanine aminotransferase, U/L	37	10–42
Ferritin, ng/mL	1563	17.9–464
Fibrinogen, mg/dL	>900	200–400
D-dimer, ng/mL	5.7	<1
Creatine phosphokinase, U/L	37	59–248
Troponin T, ng/mL	0.861	<0.014
B-type natriuretic peptide, pg/mL	>2000	<18.4

The patient's circulation stabilized, and the swollen cervical lymph nodes improved a few days after ICU admission. During his stay in the ICU, we observed generalized edema. However, as inflammation improved, his urine volume increased, and the edema improved. We observed conjunctivitis 8 days after admission. No skin rash or desquamation was observed. Echocardiography performed 11 days after admission showed improvement in cardiac contraction to 64%, and the duration of fever >38°C was 8 days.

Cultures of blood collected at admission yielded negative results. Coronary computed tomography angiography showed no aneurysms or other abnormalities in the coronary arteries.

The case definition of MIS-A in the Centers for Disease Control and Prevention report (*4*) lists the following 5 criteria: 1) severe illness requiring hospitalization in a person >21 years of age; 2) a positive test result for current or previous SARS-CoV-2 infection (nucleic acid, antigen, or antibody) during admission or in the previous 12 weeks; 3) severe dysfunction of >1 extrapulmonary organ systems (e.g., hypotension or shock, cardiac dysfunction, arterial or venous thrombosis or thromboembolism, or acute liver injury); 4) laboratory evidence of severe inflammation (e.g., elevated C-reactive protein, ferritin, D-dimer, or interleukin-6); and 5) absence of severe respiratory illness (to exclude patients in which inflammation and organ dysfunction might be attributable simply to tissue hypoxia). This case meets all of these criteria.

Whether MIS-A is associated with acute SARS-CoV-2 infection or is a reaction after acute infection is unclear. In this case, the case-patient’s positive SARS-CoV-2 test result occurred 37 days before the onset of MIS-A, and IgG levels were already elevated at the time of admission. This fact supports the notion that MIS-A can occur after the acute phase of SARS-CoV-2 infection. The only symptom at the time of infection was olfactory disturbance, which is similar to other case reports of MIS-A occurring in asymptomatic or minimally symptomatic patients (*5*).

It has been reported that MIS-A can cause symptoms similar to those of Kawasaki disease (*6*). This case did not meet the American College of Cardiology criteria for Kawasaki disease (*7*) but did meet the definition of incomplete Kawasaki disease. Conjunctivitis persisted for 4 weeks after the onset of MIS-A and gradually improved.

In February 2021, a case definition was proposed for reporting cases of multisystem inflammatory syndrome in adults and children after vaccination (*8*). Considering the possibility that the disease develops after asymptomatic SARS-CoV-2 infection and that increased IgG levels can be involved, MIS-A is rare, but the disease concept of MIS-A should be widely acknowledged. Clinicians should consider obtaining detailed history and examining SARS-CoV-2 IgG levels for cases of severe inflammatory disease with unexplained cardiac decompensation.
